# Prenatal phenotyping: A community effort to enhance the Human Phenotype Ontology

**DOI:** 10.1002/ajmg.c.31989

**Published:** 2022-07-24

**Authors:** Ferdinand Dhombres, Patricia Morgan, Bimal P. Chaudhari, Isabel Filges, Teresa N. Sparks, Pablo Lapunzina, Tony Roscioli, Umber Agarwal, Shagun Aggarwal, Claire Beneteau, Pilar Cacheiro, Leigh C. Carmody, Sophie Collardeau‐Frachon, Esther A. Dempsey, Andreas Dufke, Michael Henri Duyzend, Mirna el Ghosh, Jessica L. Giordano, Ragnhild Glad, Ieva Grinfelde, Dominic G. Iliescu, Markus S. Ladewig, Monica C. Munoz‐Torres, Marzia Pollazzon, Francesca Clementina Radio, Carlota Rodo, Raquel Gouveia Silva, Damian Smedley, Jagadish Chandrabose Sundaramurthi, Sabrina Toro, Irene Valenzuela, Nicole A. Vasilevsky, Ronald J. Wapner, Roni Zemet, Melissa A Haendel, Peter N. Robinson

**Affiliations:** ^1^ Sorbonne University, GRC26, INSERM, Limics, Armand Trousseau Hospital, Fetal Medicine Department, APHP Paris France; ^2^ American College of Medical Genetics and Genomics, Newborn Screening Translational Research Network Bethesda Maryland USA; ^3^ Institute for Genomic Medicine Nationwide Children's Hospital Columbus Ohio USA; ^4^ University Hospital Basel and University of Basel, Medical Genetics Basel Switzerland; ^5^ Department of Obstetrics, Gynecology, & Reproductive Sciences University of California, San Francisco San Francisco California USA; ^6^ CIBERER and Hospital Universitario La Paz, INGEMM‐Institute of Medical and Molecular Genetics Madrid Spain; ^7^ Neuroscience Research Australia (NeuRA), University of New South Wales Sydney New South Wales Australia; ^8^ Department of Maternal and Fetal Medicine Liverpool Women's NHS Foundation Trust Liverpool UK; ^9^ Department of Medical Genetics Nizam's Institute of Medical Sciences Hyderabad Telangana India; ^10^ Service de Génétique Médicale, UF 9321 de Fœtopathologie et Génétique, CHU de Nantes Nantes France; ^11^ William Harvey Research Institute Queen Mary University of London London UK; ^12^ Department of Genomic Medicine The Jackson Laboratory Farmington Connecticut USA; ^13^ Department of Pathology University Hospital of Lyon and Soffoet Lyon France; ^14^ St George's University of London, Molecular and Clinical Sciences Research Institute London UK; ^15^ University of Tübingen, Institute of Medical Genetics and Applied Genomics Tübingen Germany; ^16^ Department of Pediatrics Boston Children's Hospital Boston/Cambridge Massachusetts USA; ^17^ Sorbonne University, INSERM, LIMICS Paris France; ^18^ Department of Obstetrics and Gynecology Columbia University Irving Medical Center New York New York USA; ^19^ Department of Obstetrics and Gynecology University Hospital of North Norway Tromsø Norway; ^20^ Department of Medical Genetics and Prenatal diagnosis Children's University Hospital Riga Latvia; ^21^ Department of Obstetrics and Gynecology University of Medicine and Pharmacy Craiova Craiova Dolj Romania; ^22^ Department of Ophthalmology Klinikum Saarbrücken Saarbrücken Saarland Germany; ^23^ Department of Biochemistry and Molecular Genetics University of Colorado Anschutz Medical Campus Aurora Colorado USA; ^24^ Azienda USL‐IRCCS di Reggio Emilia Medical Genetics Unit Reggio Emilia Italy; ^25^ Genetics and Rare Diseases Research Division, IRCCS Ospedale Pediatrico Bambino Gesù Rome Italy; ^26^ Vall d'Hebron Hospital Campus, Maternal & Fetal Medicine Barcelona Spain; ^27^ Hospital Santa Maria, Serviço de Genética, Departamento de Pediatria Hospital de Santa Maria, Centro Hospitalar Universitário Lisboa Norte, Centro Académico de Medicina de Lisboa Lisboa Portugal; ^28^ Hospital Vall d'Hebron, Clinical and Molecular Genetics Area Barcelona Spain; ^29^ Department of Molecular and Human Genetics Baylor College of Medicine Houston Texas USA

**Keywords:** HPO, human phenotype ontology, GA4GH Phenopacket, prenatal diagnosis, fetal pathology, prenatal phenotyping

## Abstract

Technological advances in both genome sequencing and prenatal imaging are increasing our ability to accurately recognize and diagnose Mendelian conditions prenatally. Phenotype‐driven early genetic diagnosis of fetal genetic disease can help to strategize treatment options and clinical preventive measures during the perinatal period, to plan in utero therapies, and to inform parental decision‐making. Fetal phenotypes of genetic diseases are often unique and at present are not well understood; more comprehensive knowledge about prenatal phenotypes and computational resources have an enormous potential to improve diagnostics and translational research. The Human Phenotype Ontology (HPO) has been widely used to support diagnostics and translational research in human genetics. To better support prenatal usage, the HPO consortium conducted a series of workshops with a group of domain experts in a variety of medical specialties, diagnostic techniques, as well as diseases and phenotypes related to prenatal medicine, including perinatal pathology, musculoskeletal anomalies, neurology, medical genetics, hydrops fetalis, craniofacial malformations, cardiology, neonatal‐perinatal medicine, fetal medicine, placental pathology, prenatal imaging, and bioinformatics. We expanded the representation of prenatal phenotypes in HPO by adding 95 new phenotype terms under the *Abnormality of prenatal development or birth* (HP:0001197) grouping term, and revised definitions, synonyms, and disease annotations for most of the 152 terms that existed before the beginning of this effort. The expansion of prenatal phenotypes in HPO will support phenotype‐driven prenatal exome and genome sequencing for precision genetic diagnostics of rare diseases to support prenatal care.

## 1 INTRODUCTION

Genetic diagnosis can define fetal prognosis, inform parental decision‐making, and guide clinical management during pregnancy and the perinatal period. Congenital anomalies affect 2–4% of all infants and are responsible for 20% of perinatal deaths (Osterman, Kochanek, MacDorman, Strobino, & Guyer, 2015). The most recent systematic review of prenatal genomic testing with Exome Sequencing (ES) and/or Genome Sequencing (GS), identified 72 reports from 66 studies involving 4,350 fetuses with a pooled diagnostic yield of 31% (Mellis, Oprych, Scotchman, Hill, & Chitty, 2022). This represents a compelling justification for the adoption of genomic diagnostics into fetal medicine, but there is still significant room for improvement to facilitate clinical use.

The Human Phenotype Ontology (HPO) is a rich representation of abnormal human phenotypic features including signs, symptoms, laboratory test results, imaging findings, and other phenotypic abnormalities. HPO is logically structured as an ontology and enables sophisticated algorithms that support combined genomic and phenotypic analysis in diverse clinical and research applications. Examples include genomic interpretation for diagnostics, gene‐disease discovery, mechanism discovery, and Electronic Health Record (EHR) cohort analytics—all of which assist in realizing the promise of precision medicine (Haendel, Chute, & Robinson, 2018). The HPO team has developed free and open community resources consisting of the ontology itself and a comprehensive corpus of phenotype annotations (HPOA) corresponding to each of over 8,400 rare diseases. HPO has become a global standard for the International Rare Disease Research Consortium (IRDiRC) (Lochmüller et al., 2017) and has been translated into more than 10 languages for use in rare disease diagnostic tools (Köhler et al., 2018). Almost all clinical genetics diagnostic tools now leverage the HPO to encode and compute over patient features in the context of genomic variant classification. Further, by enabling data exchange, HPO unites an ever‐growing community of diverse users and contributors across the world, illuminating the natural history of disease, revealing new diseases, supporting patient registries and n‐of‐1 matchmaking for diagnosis, as well as numerous national genomic initiatives including the 100,000 Genomes Project, the NIH Undiagnosed Diseases Program and Network, the Deciphering Developmental Disorders study, and biobanking programs (Köhler et al., 2021; Robinson et al., 2008). It has also supported the discovery of genes associated with both previously known and novel human diseases by comparing HPO terms and those of the Mammalian Phenotype Ontology (MP) (Cacheiro et al., 2020; Oellrich, Hoehndorf, Gkoutos, & Rebholz‐Schuhmann, 2012).

Accurate and comprehensive phenotyping matters for accurate diagnosis. Many ES and GS pipelines incorporate phenotype analysis into approaches for ranking and interpreting variants, and having a standardized computational resource such as HPO is key to sharing and analyzing both prenatal and postnatal features. Since its inception in 2008, the majority of work on the HPO has been dedicated to describing postnatally observed phenotypes. With the advent of sophisticated methods for prenatal phenotyping and recent successes in the application of next‐generation sequencing including ES and GS, an opportunity has arisen to apply HPO‐based, phenotype‐driven, genomic analysis to prenatal sequencing. For this reason, a group of specialists in multiple disciplines related to prenatal medicine came together over a period of 2 years to extend HPO resources to better cover the prenatal phenotype. This article describes the result of this work and provides guidelines for using HPO for prenatal phenotyping.

### 1.1 Prenatal phenotyping

Common noninvasive prenatal evaluations in the first trimester include first trimester screening (FTS), noninvasive prenatal testing (NIPT) on cell‐free DNA (cfDNA) in maternal serum, and ultrasound for nuchal translucency (NT) measurement and morphological assessment of the fetus. As screening tests, these are designed to identify women with pregnancies at high risk for chromosomal and genetic conditions and congenital anomalies (Carlson & Vora, 2017). The FTS for aneuploidy risk assessment generally comprises a multiparameter algorithm including maternal age, serum screening (usually pregnancy‐associated plasma protein A and free beta‐human chorionic gonadotropin), and ultrasonographic measurement of the NT and the crown‐rump length (CRL) performed between 11 and 13 weeks 6 days gestational age (Salomon et al., 2013).

The morphological assessment of the fetus is based on ultrasound examination. High resolution ultrasound can potentially detect the majority of severe or lethal fetal structural anomalies in the first trimester scan (Blaas, 2014; Rossi & Prefumo, 2013). In most countries, a second trimester detailed anomaly scan between 18 and 22 weeks' gestation (WG) is standard of care, and with the advances of high resolution ultrasound technologies, it allows detection of the majority of fetal anomalies and assessment of the fetal phenotype at this developmental stage with high precision. Resources available for following up on potentially abnormal findings continue to improve and additional modalities, including magnetic resonance imaging (MRI), computerized tomography (CT‐Scan), and 3D/4D ultrasound, are entering the prenatal imaging space (Gray, Wilkins‐Haug, Herrig, & Vora, 2019). The catalog of prenatal phenotypes in HPO, therefore, needs to be extended to include data from these modalities.

There are a number of challenges to fetal phenotypic assessment. Prenatal phenotypes are generally less well characterized than postnatal phenotypes; further, there is a clear difficulty in identifying some organs in the prenatal period (e.g., the skin) and some anatomical structures change significantly in the postnatal period (e.g., the lungs, the cardiovascular system). Fetal phenotypes are observed indirectly by imaging modalities and the clinical information inferred from prenatal imaging is sparse in comparison with that obtained through clinical, behavioral, functional, and pathological phenotyping of the infant after delivery (Filges & Friedman, 2015). It is substantially more difficult and in some cases impossible in the prenatal period to assess functional anomalies such as seizures, developmental delay, or hearing loss. This is reflected in our assessment that only about 10% of HPO terms are identifiable through imaging modalities in the prenatal setting. A review of HPO terms with a focus on their “identifiability” during prenatal life has confirmed that only a minority are detectable with high sensitivity. Therefore, fetal phenotypes may differ from those classically associated with a newborn, child or adult with the same genetic condition. In addition, a significant proportion of abnormal phenotypes encountered during the antenatal developmental stages may be specific to fetal life since they will lead to embryonic, fetal or perinatal lethality and will have escaped etiological research and clinical delineation so far. This is epitomized by fetal Noonan syndrome where, in the second trimester fetus, pleural effusions and polyhydramnios are common, and the typical pulmonic stenosis is not easily ascertained. This was also noted in a cohort of 246 stillborn probands evaluated using ES in which there was an enrichment of loss of function (LOF) variants in genes intolerant to such variation in humans; the LOF variants were was concentrated in genes not previously associated with human disease and 44% of the candidate genes were embryonic lethal in mice (Stanley et al., 2020). Fetal phenotypes may also represent an incomplete or severe allelic presentation of a phenotype described to occur postnatally, and the diagnosis therefore can remain unrecognized at this stage of development (Filges & Friedman, 2015). Recent studies on GS/ES amongst sick infants in ICU showed that in a significant proportion of cases an abnormal prenatal phenotype would not be expected in cases where a genomic etiology was identified postnatally (Kingsmore et al., 2019; Meng et al., 2017).

Fetal phenotypes may also evolve over time since the occurrence of symptoms may be specific to the developmental stage (Mone et al., 2021a). New phenotypes can be observed with increasing gestational age, and phenotypes can resolve (i.e., cystic hygromas or hydrops fetalis may resolve) even in the presence of an underlying genetic disease. A recently published systematic review has shown that during routine third‐trimester ultrasound, an incidental fetal anomaly will be found in about 1 in 300 scanned women. This not only includes anomalies missed at the earlier 20 weeks scan but also abnormalities that can only be seen with fetal maturation, such as certain malformations of cortical development, microcephaly, or hydrocephalus; some congenital heart defects; gastrointestinal abnormalities relating to intestinal obstruction; urinary tract abnormalities that change over time such as renal pelvis dilatation; and some skeletal dysplasias (Drukker et al., 2021, Mone et al., 2021d). Tracking and real‐time updating of the fetal phenotype in combination with the application of genomic testing in prenatal diagnosis has become an active field of research. As a consequence, HPO can support this effort by curation of prenatal phenotypes as available. For example, a finding in the fetal period may sometimes suggest a specific disease and the same finding in a child or adult may not be related to the same disease. This is the case of cardiac rhabdomyomas: when observed in the fetal age they suggest the diagnosis of tuberous sclerosis but when observed in teenagers or adults, alternative diagnoses are more likely (Boitor Borza, Popa Stanila, Zaharie, Hasmasanu, & Muresan, 2022).

In summary, fetal phenotypes of genetic diseases are often unique and at present are not well understood. We lack knowledge of the natural history of most genetic diseases in the prenatal period. Certain manifestations can occur prenatally but resolve or evolve postnatally. For instance, fetal effusions, polyhydramnios, and contractures can be prenatal manifestations of RASopathies but are not postnatal features of these diseases. In order to extend our understanding of these diseases and their prenatal manifestations, it is essential to create HPO resources to allow comprehensive and precise computational models of fetal phenotypes and to link these features to fetal manifestations of disease.

### 1.2 Prenatal genetic and genomic diagnosis (PD)

In contrast to screening approaches, PD provides diagnostic information as to the etiology and prognosis as well as informing pregnancy, perinatal and neonatal decision‐making and management. Fetal aneuploidy and unbalanced chromosomal rearrangements can be identified in about 35% of structurally abnormal fetuses, and chromosomal microarray (CMA) analysis identifies copy number variants in an additional 3–6.5% of cases (Hillman et al., 2013; Wapner et al., 2012). Altogether, these tests identify a genetic etiology in up to 40% of abnormal fetuses. Until recently, published experience on the diagnostic yield of prenatal ES/GS was limited to relatively small cohorts but suggested the ability of ES/GS to diagnose monogenic disorders in fetuses with normal CMA analysis. Retrospective studies for selected indications and in small patient series using various sequencing approaches show diagnostic yields between 6.5% and 80% (Best et al., 2018). Targeted exome testing on fetuses with a suspected skeletal dysplasia phenotype showed a diagnostic rate of 81% (Chandler et al., 2018), and in some cases ES/GS reduced morbidity and lowered inpatient costs (Clark et al., 2018; Farnaes et al., 2018; Talkowski et al., 2012; Warburton, 1991). Two large prospective studies that applied ES to large cohorts of fetuses with structural anomalies were performed in cases where CMA was unrevealing. One prospective study of 610 fetuses with structural anomalies (PAGE) identified a clinically significant genetic variant in 8.5% of cases (Lord et al., 2019). In the second study, a diagnostic variant was found in 10.3% of fetuses (Petrovski et al., 2019). Both studies and subsequent studies showed that the diagnostic yield was higher in fetuses with multiple congenital anomalies, skeletal dysplasias and cardiac anomalies (Lord et al., 2019; Mone et al., 2021c, 2021e; Petrovski et al., 2019). In 3.2% of pregnancies with an isolated increased first trimester nuchal translucency, monogenic etiologies were identified as compared with 25% of pregnancies with nonimmunological hydrops. Pathogenic variants in genes of the RASopathy pathways seem to account for the majority of such severe phenotypes (Mone, Eberhardt, Hurles, et al., 2021c, 2021e; Sparks et al., 2020). When including deep phenotyping throughout pregnancy and postnatally on the initial patients of the PAGE cohort, a genetic diagnosis was obtained in over half of ultrasound detected fetal structural anomalies (Mone, Abu Subieh, Doyle, et al., 2021a). A systematic review and meta‐analysis of 66 ES/GS studies in fetal structural anomalies representing 4,350 fetuses concluded that prenatal exome sequencing provides an additional diagnostic yield of 31% after normal CMA (Mellis et al., 2022). The diagnostic yield was reported to be significantly higher in clinically pre‐selected cases (42% vs. 15%) and differed between phenotypic subgroups ranging from 53% for isolated skeletal abnormalities to 2% for isolated nuchal translucency (Mellis et al., 2022).

### 1.3 HPO terms and annotations

**FIGURE 1:**
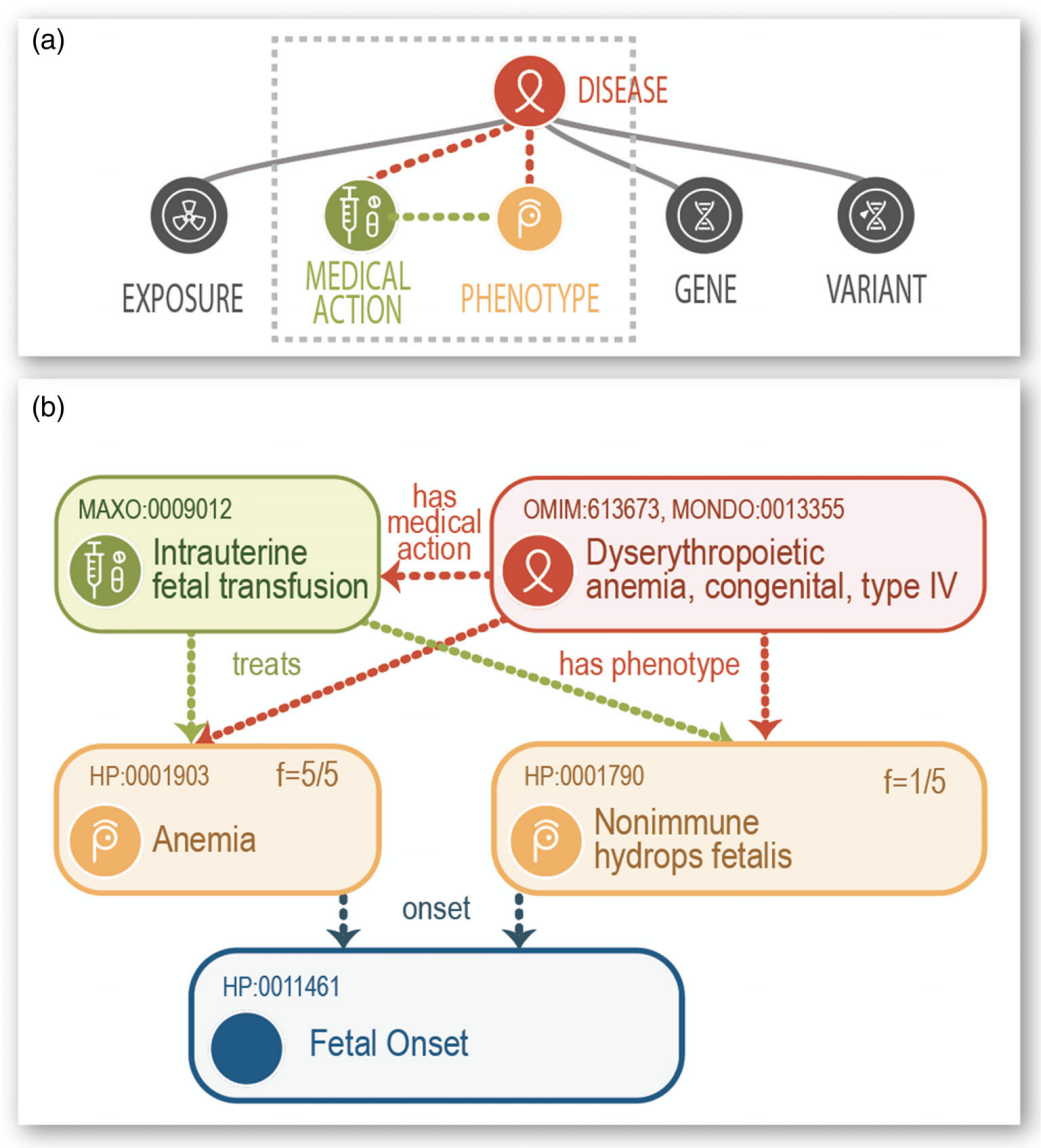
Example attributes of the HPO disease annotation model (HPOA). (a) Schematic representation of our model of a rare disease. Associated with the diseases are a variety of features. (b) This schematic represents one HPO annotation for the disease dyserythropoietic anemia, congenital, type IV. Published phenotypes include anemia with infantile onset; all five of the patients with the disease presented this way (shown as *f* = 5/5). An annotation to a treatment (intrauterine fetal transfusion coded by a MAxO term) is shown; the treatment treats anemia, as well as nonimmune hydrops fetalis, a less frequent phenotypic manifestation of the disease.

In addition to the ontology itself, the HPO project provides HPO annotations (HPOAs) of diseases. For instance, the disease *Marfan syndrome* is characterized by—and therefore annotated to—over 50 phenotypic abnormalities including *Aortic aneurysm* (HP:0004942) (each abnormality is represented by an HPO term). The annotations can have modifiers that describe the age of onset and the frequencies of features. For instance, the phenotypic abnormality *Brachydactyly* (HP:0001156) is rare in *Hydrolethalus syndrome* (3/56 according to a published study referenced in our data), but affects nearly 100% of patients diagnosed with most of the 484 other diseases annotated to this term. This type of information can be used by algorithms to weight findings in the context of clinical differential diagnosis. The Monarch Initiative is additionally creating the Medical Action Ontology (MAxO), a new ontology of clinically relevant actions (e.g., treatments and interventions) to integrate these actions into the HPO disease models for use in clinical decision‐making when interpreting variants and performing diagnostics for RDs. A full disease model includes phenotypic features, links to genes and variants, a representation of treatments and other actions, and (if relevant) environmental exposures (Figure 1).

The description of phenotypic variation has become central to translational research and genomic medicine (Biesecker, 2004; Deans et al., 2015; Robinson, 2012; Robinson & Webber, 2014), and computable descriptions of human disease using HPOAs have become key to a number of genomic diagnostic algorithms. Our group has developed HPO‐based software for genomic diagnostics called Exomiser/Genomiser (Robinson et al., 2014; Smedley et al., 2015, 2016) that is widely used by projects such as the NIH Undiagnosed Diseases Program (Bone et al., 2016; Gall et al., 2017) and Genomics England's 100,000 Genomes project (100,000 Genomes Project Pilot Investigators et al., 2021) (Chief Medical Officer annual report, 2016: Generation Genome). The HPO allows algorithms to “compute over” clinical phenotype data in a wide variety of contexts. The ontological structure of the HPO allows fuzzy phenotype matching (semantic similarity) of sets of individual terms (phenotypic profiles, encoded within HPOAs) (Bauer, Köhler, Schulz, & Robinson, 2012; Köhler et al., 2009; Schulz, Köhler, Bauer, Vingron, & Robinson, 2011). Additionally, the underlying logical definitions enable HPO terms to be integrated with numerous other resources, such as model organism data (Doelken et al., 2013; Köhler et al., 2013, 2014; Mungall et al., 2015; Robinson & Webber, 2014; Washington et al., 2009).

### 1.4 Prenatal extension of the HPO

The HPO consortium has periodically organized collaborative workshops to revise and extend specific areas such as ophthalmology, immunology, nephrology, and neurology (Lewis‐Smith et al., 2021; Haimel et al., 2021; Ong et al., 2020; Gasteiger et al., 2020; Köhler et al., 2017, 2019). In 2020, we began a series of workshops to expand the representation of phenotypic abnormalities that are observed in the prenatal period. Because of the COVID‐19 pandemic, the workshops were conducted by videoconferencing. Participants included experts in perinatal pathology, musculoskeletal anomalies, neurology, medical genetics, hydrops fetalis, craniofacial malformations, cardiology, neonatal‐perinatal medicine, fetal medicine, informatics, placental pathology, and prenatal imaging.

Some phenotypic features are only observable in the prenatal period. The HPO groups such terms under a dedicated subontology that descends from the grouping term *Abnormality of prenatal development or birth* (HP:0001197). Other features such as *Preaxial polydactyly* (HP:0100258) can be ascertained both pre‐ and postnatally. The HPO puts such terms underneath the main hierarchy (for instance, the path from *Preaxial polydactyly* to the root of the ontology is *Preaxial polydactyly→Polydactyly→Abnormal digit morphology→Abnormal limb bone morphology→Abnormal appendicular skeleton morphology→Abnormal skeletal morphology→Abnormality of the skeletal system→Abnormality of the musculoskeletal system→Phenotypic abnormality*). Specifically prenatal skeletal abnormalities are placed under *Abnormal fetal skeletal morphology* (HP:0025662), such as *Hypoplastic nasal bone* (HP:0025707). Because *Hypoplastic nasal bone* is a subterm (child) of *Abnormal skeletal morphology*, it is included by searches and phenotypic similarity algorithms such as LIRICAL under both skeletal abnormalities as well as specifically prenatal abnormalities.

Ongoing work is adding annotations to terms that specify the diagnostic modalities by which a phenotypic abnormality can be ascertained. For instance, *Inferior crossed fused renal ectopia* (HP:0034230) is annotated via the relation “is observable through” to the Medical Action Ontology (MAxO) term *prenatal renal ultrasonography* (MAXO:0009009). When this work is finished, software will be able to use the annotations to suggest HPO terms and the investigations by which they can be ascertained that are most helpful given a certain differential diagnosis and age of the patient. Additionally, the SUOG European project (www.suog.org) aims to associate fetal ultrasound phenotypes with specific ultrasound planes (or views) and other technical elements (2D, 3D; Doppler mode) to augment the ultrasound examination with the support of semantically derived imaging protocols (Dhombres et al., 2019).

The HPO prenatal workshops added 95 terms to the *Abnormality of prenatal development or birth* (HP:0001197) sub‐hierarchy (for a total of 247) and revised definitions, synonyms, and disease annotations for most of the 152 terms existing before the start of the workshops. 65 terms descend from *Abnormalities of placenta or umbilical cord* (HP:0001194). Many additional terms were discussed on the GitHub tracker of the HPO project, which currently lists 91 issues with label prenatal/perinatal/neonatal that pertain to term requests for both specifically prenatal features as well as other features that can be observed pre‐ and postnatally (April 14, 2022 release; 86 issues have been closed).

### 1.5 Age of onset

**FIGURE 2:**
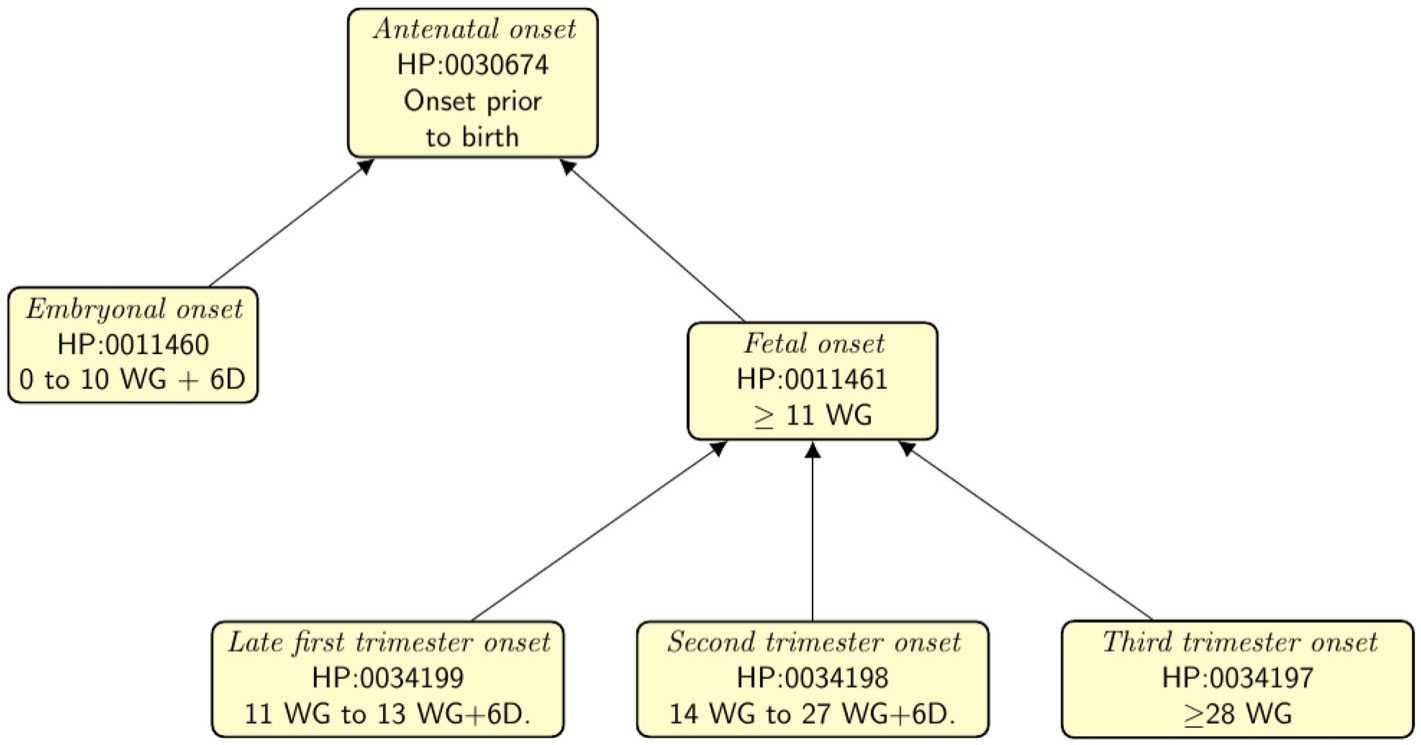
Prenatal onset terms. The HPO provides terms to denote the range of onset of diseases or phenotypic features. New terms were added for more granularity in the prenatal period. Prenatal ages are shown as gestational weeks, which are defined as the time from the last menstrual period of the mother. For instance, embryonal onset refers to a disease or phenotypic feature that is first observed in the 8 weeks following fertilization, which corresponds to 10 weeks of gestation (WG). 13 WG + 6D refers to a gestational age of 13 weeks and 6 days.

The HPOA disease annotations include in some cases information about the age of onset of a disease or of a specific phenotypic feature of the disease. These annotations are intended to specify the range of ages in which individuals with the disease in question tend to develop clinical manifestations, ranging from *Antenatal onset* (Onset prior to birth; HP:0030674), to *Late onset* (onset of symptoms after the age of 60 years; HP:0003584). As a part of the series of prenatal workshops, additional terms were added to the *Antenatal onset* subhierarchy (Figure 2). As gestational age (GA) is described in weeks from the last menstrual period (LMP or WG) during ultrasound examination, we decided to keep this for consistency with ultrasound phenotypes: for example, 8 weeks of embryonic development correspond to 10 WG.

### 1.6 HPO‐based Phenopackets for computable case reports

The Global Alliance for Genomics and Health (GA4GH) is developing a suite of coordinated standards for genomics for healthcare (Rehm et al., 2021). The Phenopacket is a new GA4GH standard for sharing disease and phenotype information. A Phenopacket characterizes an individual person or biosample, linking that individual to detailed phenotypic descriptions, genetic information, diagnoses, and treatments. The Phenopacket schema enables comparison of sets of phenotypic attributes from individual patients. Such comparisons can aid in diagnosis and facilitate patient classification and stratification for identifying new diseases and treatments (Jacobsen et al., 2021).

The HPO disease annotations are computational models of disease with information about the full range of phenotypic abnormalities and in many cases the frequencies and typical ages of onset of specific phenotypic features in cohorts of individuals with the disease (Köhler et al., 2021). In contrast, a Phenopacket represents an individual case report. Phenopackets have been used to support rare‐disease diagnostics in the SOLVE‐RD project of the European Union (Zurek et al., 2021) and can be used to support a number of additional applications in translational research and computational decision support. Several tools for phenotype‐driven genomic diagnostics already support the Phenopacket Schema, including Exomiser, LIRICAL, Phen2Gene, and CADA (Peng et al., 2021; Robinson et al., 2014, 2020; Zhao et al., 2020), and we anticipate that more tools will offer Phenopacket support in the future.

**FIGURE 3:**
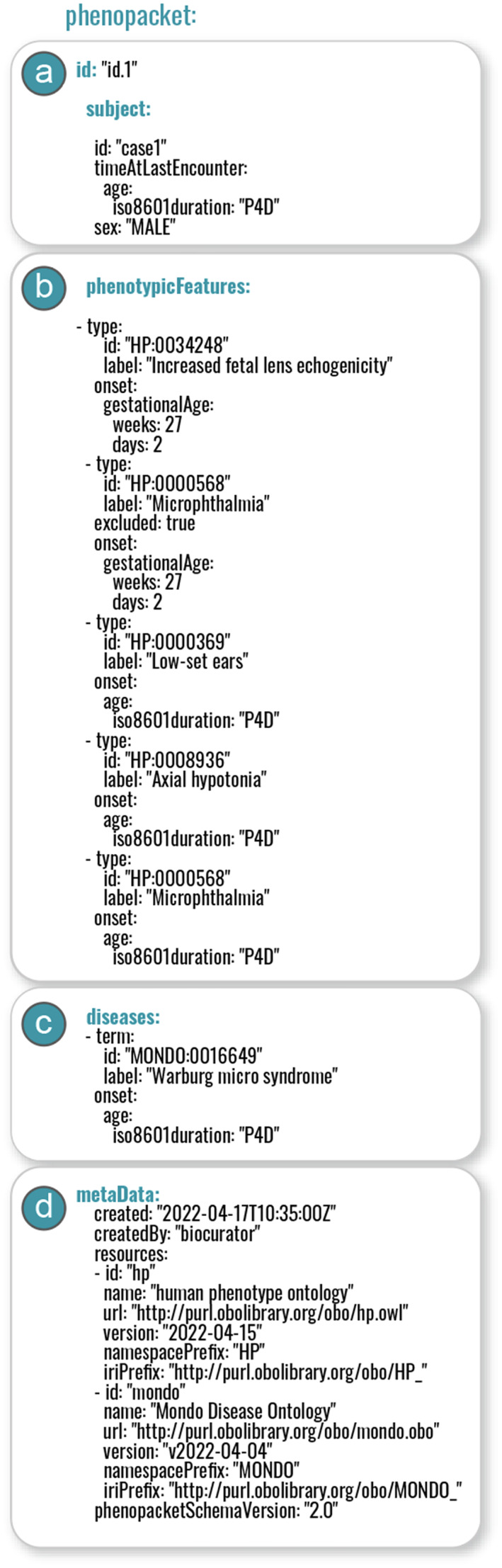
Components of a Phenopacket. (a) The subject message provides basic demographic information; (b) The phenotypicFeatures message consists of a list of phenotypic features (each of which begins with “‐type” in YAML format as shown here). The patient was found to have *Increased fetal lens echogenicity* (HP:0034248) at a gestational age of 27 weeks, at which time *Microphthalmia* (HP:0000568) was excluded. Postnatal examination at an age of 4 days (represented in iso8601 format as P4D) shows *Axial hypotonia* (HP:0008936), *Low‐set ears* (HP:0000369), and *Microphthalmia* (HP:0000568). (c) The disease diagnosis is recorded using a term from the Mondo ontology (Shefchek et al., 2020), *Warburg micro syndrome* (MONDO:0016649). (d) The metaData message records versions of ontologies used to create the Phenopacket together with other information. Additionally, the Phenopacket schema provides resources to record measurements, biospecimens, and treatments, not shown here.

Here we show how a Phenopacket might be used to encode clinical data to support exome or genome analysis. We have adapted a case of fetal cataract in which subsequent postnatal investigations revealed additional phenotypic abnormalities that led to a diagnosis of Warburg micro syndrome (Léonard, Bernard, Hiel, & Hubinont, 2009). Figure 3 provides an explanation and we refer to the online documentation for technical details (Phenopacket‐schema 2.0 documentation). In this example, the Phenopacket provides additional context for the phenotypic abnormalities that goes beyond what could be provided by a simple list of HPO terms. For instance, the age of onset of each feature is provided, and the fact that Microphthalmia was excluded on prenatal sonography is indicated. For prenatal features, the precise age of onset is often difficult to determine, and the “onset” field should be used to report the age of first observation.

As a first application of Phenopacket schema for prenatal medicine, the HPO team is collaborating with the Fetal Sequencing Consortium (FSC; Giordano & Wapner, 2022). The FSC includes >30 national and international sites involved in fetal sequencing whose members participate in bi‐weekly calls to share data, challenges, and discuss new technologies relevant to sequencing in the perinatal period. The FSC has sequenced >3,000 anomalous and stillbirth probands, finding that 13–26% of fetuses with structural anomalies currently have a causative genotype using American College of Medical Genetics (ACMG) guidelines.

This effort has expanded to the Fetal Genomics Consortium (FGC), with a mission to understand the genetic and phenotypic architecture of fetal anomalies and stillbirth and use this information to inform pre‐, peri ‐, postnatal and maternal care. Phenotypic and genotypic data from across the FGC centers will be integrated, aggregated, and analyzed. This collaboration will leverage the Terra Platform (https://terra.bio/), a cloud‐based data ecosystem enabling processing and analysis of genomic data. Integrating phenotype data into analysis in Terra is currently limited, and a goal is to build‐out the ability to easily store and run analysis using Phenopackets and associated tools in Terra. This would, for the first time, enable truly integrated genotype and phenotype analyses in one cloud‐based platform that is accessible to researchers and clinicians worldwide.

We anticipate that the phenotype‐driven analysis will require tuning to particular cohorts, and working with the FGC will: (1) drive new systematic phenotypes in the prenatal space; (2) allow benchmarking of phenotypic driven analysis in a single, large cohort; and (3) enable phenotype driven analysis on an existing, widely used platform, Terra.

## 2 DISCUSSION

We have presented the work of a group of experts in various fields of fetal medicine to extend the HPO to cover the prenatal phenotypic manifestations of disease. The majority of rare genetic diseases have good phenotypic descriptions and consequently HPOA in children and adults, available in gene‐centric form from the HPO consortium and in disease‐centric form from Orphanet (Maiella et al., 2018). However, scant phenotypic data are available for the prenatal period. Prenatal findings of some genetic diseases have only recently begun to be collected in the literature (García‐Santiago et al., 2021; Sarac Sivrikoz et al., 2022), and these annotations have been transferred more slowly to the databases, limiting their computational management. For this reason, the HPO prenatal team has made an effort to include new prenatal HPO terms to support rapid inclusion in databases. Also, detailed ultrasound findings from the ontology developed by the SUOG consortium will continue to support the extension of prenatal HPO terms.

To take full advantage of ontology‐based algorithms for translational research in fetal medicine and variant interpretation in prenatal ES/GS analysis, additional clinical data, computational resources, and algorithms will be required. A paucity of data is available about the prenatal manifestations of most Mendelian diseases, and many relevant articles are reports about single cases that are insufficient to understand the scope of variability and the natural history of diseases. Efforts such as the FGC can help distribute datasets in a federated fashion; the use of the GA4GH Phenopacket to code each case in an interoperable and computable fashion should accelerate the computational use of case‐level data. Journals in the field are to be encouraged to have authors submit Phenopackets as supplemental material with case reports and articles about cohorts of patients. Innovative ways of encoding phenotype data as a part of the clinical encounter are imaginable. For instance, the fetal femur length is measured as a part of some fetal sonographic examinations. Knowledge of the femur length, the sex and gestational age of the fetus would allow a simple algorithm to determine if *Short fetal femur length* (HP:0011428) is present or not, which would allow HPO terms to be immediately generated as a part of the fetal sonography evaluation. Furthermore, phenotypes may be refined with new imaging modalities, such as fetal MRI or fetal CT‐scan. Comprehensive aggregation of prenatal phenotype and genotype data will permit expansion of the phenotypic spectrum of a disorder, and inform both pre‐ and postnatal diagnosis.

In humans, information on genes associated with prenatal and neonatal phenotypes is limited and/or not systematically captured in a comprehensive manner. As more prenatal data become available, algorithms for cross‐species phenotype matching may become relevant for characterization of novel associations between genes and human diseases with prenatal manifestations (Robinson & Webber, 2014). Prenatal ES studies have been successful in characterizing previously unrecognized prenatal phenotypes associated with known Mendelian disease genes, and in many cases have leveraged comparisons to embryonic phenotypes in mouse models (Filges et al., 2014; Fujikura et al., 2013; Vora et al., 2017). Large scale efforts such as the International Mouse Phenotyping Consortium (IMPC) are creating genome‐ and phenome‐wide catalogs of gene function by characterizing new knockout mouse strains across diverse biological systems through a broad set of standardized phenotyping tests (Meehan et al., 2017). Current estimates are that gene inactivation leads to lethal embryonic or perinatal phenotypes in roughly 30% of gene knockout experiments (Adams et al., 2013). An additional 13% (198/1751) are associated with subviable phenotypes in which fewer than half the expected number of homozygous pups survive (Dickinson et al., 2016). Future algorithms for variant interpretation in prenatal genomics will be able to make use of the HPO annotations and their ready comparison to data from other species that is the focus of the Monarch Initiative (Shefchek et al., 2020). Such analyses could include data on the time course of prenatal manifestations or death, and further allow discrimination between phenotypes leading to prenatal lethality. These early manifestations are part of a wider, postnatal phenotyping spectrum that can be used to improve prioritization of genes and diseases and ultimately care and family planning. Due to the rapidly growing importance of ES/GS for the diagnosis of monogenic disorders in fetal anomaly phenotypes, extension of cross‐species algorithms and tools such as Exomiser (Smedley et al., 2015) could support analysis of prenatal phenotypes in genomic diagnostics.

The HPO consortium welcomes the continued participation of the prenatal genomics community in the extension and improvement of HPO resources in this area.

## FUNDING INFORMATION

This study was supported by NHGRI [1U24HG011449‐01A1] and NIH Office of the Director 2R24OD011883‐05A1], the European Union's Horizon 2020 research and innovation program under grant agreement No. 779257 (SOLVE‐RD), The European Union's EIT‐Health Innovation Program bp2020‐2022, #20062 and #211015 (SUOG‐Smart Ultrasound in Obstetrics and Gynecology), FIS‐ISCIII PMP21/00063, the Australian Federal Government Medical Research Futures Fund (MRFF) PreGen: Filling the Gap—Antenatal Genomics and Newborn Care: The Translational PreGen Consortium and PI 20/01053, and British Heart Foundation grant FS/18/78/33932. This study has been partly generated within the European Reference Network on Rare Congenital Malformations and Rare Intellectual Disability (ERN‐ITHACA). ERN‐ITHACA is co‐funded by the Health Program of the European Union.

## Data Availability

The data that support the findings of this study are openly available in Human Phenotype Ontology at https://hpo.jax.org/app/.
